# Indocyanine green fluorescence perfusion testing in robot-assisted hepatic arterial infusion pump placement

**DOI:** 10.1007/s00464-024-11010-7

**Published:** 2024-07-17

**Authors:** Roderick W. J. J. van Dorst, Britte H. E. A. Ten Haaft, Stijn Franssen, Inne H. M. Borel Rinkes, Bas Groot Koerkamp, Rutger-Jan Swijnenburg, Jeroen Hagendoorn

**Affiliations:** 1 Department of Surgery, UMC Utrecht Cancer Center, University Medical Center Utrecht, Heidelberglaan 100, 3584CX Utrecht, The Netherlands; 2 Department of Surgery, Cancer Center Amsterdam, Amsterdam UMC, Amsterdam, The Netherlands; 3 Department of Surgery, Erasmus MC Cancer Institute, Erasmus University Medical Center, Rotterdam, The Netherlands

**Keywords:** Robot-assisted surgery, Chemotherapy, Fluorescence

## Abstract

**Background:**

Hepatic arterial infusion pump (HAIP) treatment is a technique used to treat liver localized malignancy with intra-arterial chemotherapy. Methylene blue is generally administered to verify hepatic perfusion and exclude inadvertent extrahepatic perfusion. The use of indocyanine green dye (ICG) combined with near-infrared (NIR) fluorescence imaging during robot-assisted HAIP placement may be an attractive alternative by providing high contrast without blue discoloration of the operative field.

**Methods:**

Data was collected retrospectively from 2 centers in the Netherlands. Intraoperative perfusion of the liver segments and extrahepatic perfusion were assessed using ICG/NIR as well as methylene blue on video imaging and correlated to postoperative 99 m-Tc perfusion scintigraphy.

**Results:**

13 patients underwent robot-assisted surgery for HAIP placement; median length of stay was 4 days, complications occurred in 4 patients. Hepatic perfusion showed identical patterns when ICG was compared with methylene blue. In 1 patient, additional extrahepatic perfusion was found using ICG, leading to further vessel ligation. Intraoperative ICG perfusion was concordant with 99 m-Tc perfusion scintigraphy.

**Discussion:**

Liver and extrahepatic perfusion determined by ICG fluorescence imaging is concordant with blue dye perfusion and 99 m-Tc perfusion scintigraphy. Therefore, ICG fluorescence imaging is deemed a safe and reliable technique for perfusion testing during robot-assisted HAIP placement.

**Graphical abstract:**

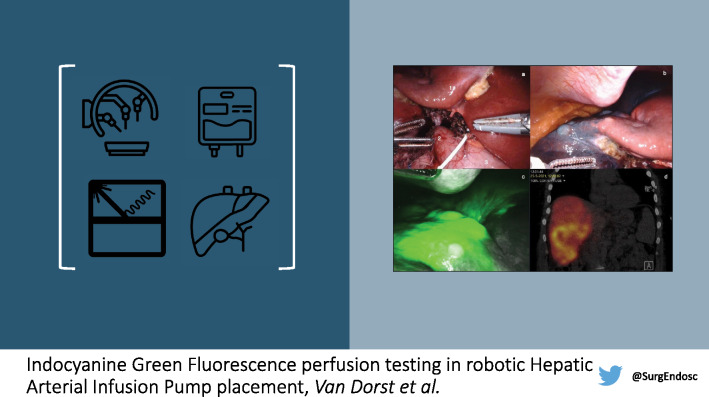

**Supplementary Information:**

The online version contains supplementary material available at 10.1007/s00464-024-11010-7.

In locally advanced or multifocal malignancy confined to the liver or bile ducts, ‘regional’ chemotherapy can offer a solution [1]. A catheter is placed in the gastroduodenal artery, and can be used to give high concentrations of floxuridine (FUDR) chemotherapy to the liver. Due to liver tumors mainly deriving their blood from the hepatic artery, this leads to high locoregional dosing, while minimizing systemic side effects of treatment due to a high first pass effect [2]. The goal of this chemotherapy is to downstage the tumor from unresectable to resectable or to optimize palliative therapy by increasing chemotherapy treatment efficacy [3]. Delivering chemotherapy using a hepatic arterial infusion pump (HAIP) is a complex process which requires a multidisciplinary expert approach. Placement of a HAIP is associated with a complication rate of 22% both short and long term [4, 5]. Whereas most published studies on HAIP therapy for colorectal liver metastases and intrahepatic cholangiocarcinoma employ HAIP placement via laparoscopy or open approach, recent reports describe minimally invasive pump placement using a surgical robot [6]. Robot-assisted surgery allows for greater range of motion of instruments, clearer and immersive 3D view for the surgeon and allows for removal of tremors. This increases surgical accuracy and ergonomics [7, 8].

One of the other advantages of robot-assisted surgery is the standard option to apply intraoperative light filters to the robot camera. Indocyanine green dye is a fluorescent dye that emits a green light when combined with near-infrared (NIR) fluorescence imaging and can be imaged in DaVinci robot-assisted surgery using Firefly mode. In robot-assisted liver surgery, ICG can be injected systemically during surgery to identify transection planes upon vascular exclusion or pre-operatively for tumor delineation [9]. An advantage of using NIR is the tissue penetration of up to 10 mm as well as providing the ability for the user to turn visual contrast on and off with the finger clutch. During HAIP placement, it is critical to ensure that the therapy arrives at the intended target in the liver, without accidentally perfusing extrahepatic tissues such as hepatic pedicle lymph nodes or duodenum via small arterial branches arising from the proper/right/left hepatic arteries such as hepatic pedicle lymph nodes or duodenum. In standard open HAIP placement, methylene blue dye is injected via the pump side port to assess (extrahepatic) perfusion. We hypothesize that, alternatively, ICG fluorescence imaging may be ideally suited to judge homogeneity of perfusion as well as potential leakage from the catheter placement site with great accuracy, as an alternative to methylene blue [10].

To date, only a single case report describing the use of ICG for HAIP perfusion testing has been published [11]. The aim of this paper is to demonstrate the feasibility and efficacy of robot-assisted HAIP placement with use of ICG fluorescence imaging for hepatic and extrahepatic perfusion testing with in-patient comparative analysis of intraoperative ICG, methylene blue and postoperative 99 m-Tc perfusion scintigraphy.

## Patients & methods

Data was gathered retrospectively from all patients that received an intrahepatic arterial pump using the DaVinci surgical robot and ICG fluorescence imaging in the University Medical Center Utrecht (UMC Utrecht) and the Amsterdam University Medical Center (Amsterdam UMC), starting in 2020 up to the end of 2022. UMC Utrecht and Amsterdam AMC are both tertiary specialized hospitals with ‘high volume’ HPB/robot-assisted surgery programs are in the Netherlands.

As HAIP therapy in the Netherlands is currently only offered within the prospective PUMP trials, patients were indicated for HAIP placement according to study protocol for adjuvant therapy following resection of colorectal liver metastasis or palliative therapy for unresectable intrahepatic cholangiocarcinoma [12], in this context, written consent and IRB approval was obtained. ICG fluorescence testing was later added to the procedure, therefore it was not consequently performed by all centers involved in the PUMP trials. HAIP placement was performed either as a solitary procedure or with concomitant hepatectomy or colectomy. In order to place the HAIP, careful dissection of the hepatoduodenal ligament and surrounding lymph nodes is required in order to dissect the gastroduodenal artery, the common hepatic artery and the proper hepatic artery. The distal end of the gastroduodenal artery is ligated and arteriotomy is performed in order to insert the HAIP catheter, which is then secured. A video showing the placement in more detail is provided in the Supplementary Materials.

**Fig. 1:**
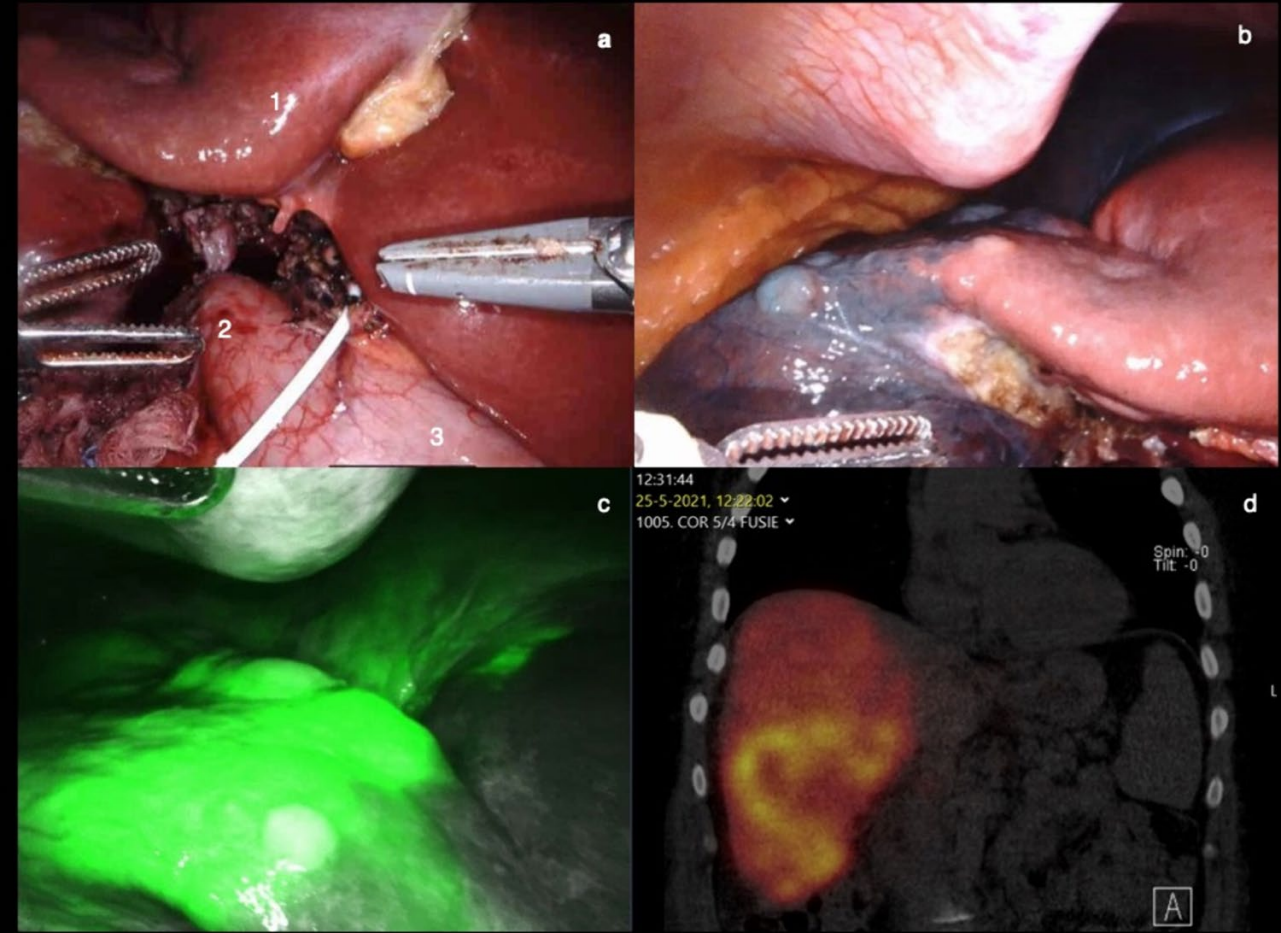
**a**: Intraoperative view (1: liver, 2: duodenum, 3: stomach), **b**: Methylene blue, **c**: ICG fluorescence, **d**: 99-Nm Technetium scintigraphy (Color figure online)

Catheter placement was inspected intraoperatively with methylene blue dye and directly after with indocyanine green dye both injected into the pump side port which is connected to the gastroduodenal artery through the catheter. For perfusion testing, 2–4 ml of ICG was administered in a 1:4 ratio with 0.9% saline solution. Inspection focused on judging perfusion of all visible liver segments as well as homogeneity of perfusion, while ensuring no extrahepatic perfusion occurs. An example of the intraoperative field and perfusion testing is shown in Fig. 1. Visual inspection of perfusion is optimal after 10 s and can be performed up to 45 s after administration, when tissue saturation is reached.

All patients remained in hospital following surgery according to local standard of care. Postoperative 99 m-Tc perfusion scintigraphy was on an in- or outpatient basis, depending on length of stay. 99 m-Tc perfusion scintigraphy is seen as the gold standard for liver perfusion testing, as shown in Fig. 1d [13]. Complications were registered as early (≤ 30 days) or late (> 30 days) and were classed using the Clavien-Dindo classification [14]. Patients were followed up to present or death. There were no cases with post-operative liver failure.

Statistical analyses were performed using the IBM Statistical Package for the Social Sciences (SPSS) version 27.0.

## Results

Patient demographics and characteristics are shown in Table 1. During the study period, thirteen patients underwent robot-assisted HAIP placement with ICG fluorescence perfusion testing. Ten of these patients primarily presented with an irresectable intrahepatic cholangiocarcinoma, the other three presented with colorectal liver metastases. The median age at time of the operation was 66 years old (51–74), eight of thirteen patients were female. Median BMI was 25 (21–36). Ten patients scored ASA 2 and the other three ASA 3. No patients had received hepatic surgery or radiation therapy prior to HAIP placement, however four patients had prior abdominal surgery. Neoadjuvant chemotherapy was given in four patients. Arterial anatomy varied greatly in patients. Six patients had normal anatomy with both left and right hepatic arteries originating from the proper hepatic artery without aberrant branches. The other seven patients had a range of variants of normal arterial anatomy. The most common variant was a replaced right hepatic artery (RHA), which occurred in four patients. Anatomic variants did not influence intra-operative decision making or outcomes.

**Table 1:** Patient demographics and pre-operative characteristics

Parameter	All patients (*n* = 13)
Age (years)	66 (51–74)
Gender, female (%)	8 (62)
BMI^1^ (kg/m^2^)	25 (21–36)
ASA^1^	
1	0
2	10
3	3
4	0
Prior abdominal surgery	4
Prior hepatic surgery	0
Prior chemotherapy	4
Prior radiation therapy	0
Arterial anatomy of hepatic artery	
Normal	6
Variant	7
Replaced RHA	4
Replaced LHA	1
Accessory LHA	1
Trifurcation of CHA	1

^1^*BMI* Body mass Index, *ASA* American society of anesthesiologists

Table 2 contains intraoperative findings and intraprocedural outcomes. During two procedures a hemicolectomy was performed concomitantly with the HAIP placement and in one case a hepatectomy was performed prior to HAIP placement. In all other ten patients the HAIP placement was the sole procedure. No conversion to open was deemed necessary and no technical problems were encountered with the device, surgical robot or near-infrared imaging. The median operative time was 235 min (172–480) including all concomitant interventions. Time spent on HAIP placement was a median of 213 min (110–310). Time spent on HAIP placement includes extensive dissection of the hepatoduodenal ligament and resection of local lymph nodes. In most patients, blood loss was negligible with only three out of thirteen patients having estimated blood loss over 100 ml. During all operations the catheter was successfully placed in the gastroduodenal artery and liver perfusion was visually inspected with methylene blue and ICG fluorescence. Extrahepatic perfusion was not detected in any patients using methylene blue, in one case a small accessory bundle to the arteria hepatica propria was found during the following inspection with ICG fluorescence, which was then ligated. In all patients the pattern of perfusion of liver segments using methylene blue and ICG was identical. The presence of variants from normal arterial anatomy did not influence intra-operative perfusion of the liver, as it is practice to ligate replaced and accessory arteries during the procedure. A video is included in the Supplementary Materials in order to illustrate perfusion testing using ICG and methylene blue as well as to show an example of the procedure.

**Table 2:** Operative findings

Parameter	All patients (*n* = 13)
Procedure	
HAIP placement	10
HAIP placement and hepatectomy	1
HAIP placement and hemicolectomy	2
Conversion to open (%)	0 (0)
Operative time (minutes)	235 (172–480)
Operative time spent on HAIP (minutes)	213 (110–301)
Estimated blood loss (ml)	4 (0–400)
Extrahepatic perfusion when inspected with methylene blue dye	0
Extrahepatic perfusion when inspected with indocyanine green dye	1
Technical problems encountered	0
Final catheter positioning in GDA (%)	13
Even liver perfusion	13

Postoperative patient outcomes are shown in Table 3. In all patients, postoperative 99 m-Tc perfusion scintigraphy showed perfusion of liver segments concordant with intraoperative methylene blue/ICG and found no extrahepatic perfusion in any patients. Median length of hospital stay was 4 days (1–14). Time to start FUDR was 14 days (11–48), in one case FUDR was not started after patient became septic and was admitted to the intensive care department. This patient underwent HAIP placement with concomitant hemicolectomy. Microbial analysis of the pump pocket fluid showed no signs of an infection, the focus was found to be anastomotic leakage of the colon. One patient complained of heavy postoperative pain which was treated conservatively. Another patient presented with fever during admission which led to discovery of an abscess in the liver which was percutaneously drained and treated with antibiotics that led to full recovery. One patient developed cholangitis after several months possibly due to stenting, treated with ursodeoxycholic acid. None of the complications were causally linked to usage of either methylene blue or ICG. Two of these complications were classed as severe (Clavien-Dindo ≥ 3a), the other two were grade 1 and 2.

**Table 3:** Patient outcomes

Parameter	All patients (*n* = 13)
Length of stay (days)	4 (1–14)
Time to start FUDR (days)	14 (11–48)
Extrahepatic perfusion postoperatively	0
Complications	4
Complications directly related to HAIP	2
Complication timing (Early ≤ 30 days)	
Abscess	1
Sepsis	1
Post-operative pain	1
Complication type (Late > 30 days)	
Cholangitis	1
Clavien Dindo ≥ 3a	2
Clavien Dindo < 3a	2

## Discussion

Indocyanine green dye in combination with near-infrared fluorescence is a feasible and safe technique to examine bilobar liver perfusion and rule out extrahepatic perfusion during HAIP placement. When testing liver and extrahepatic perfusion with ICG it is not outperformed by methylene blue in this small cohort. All intraoperative findings related to perfusion were confirmed postoperatively using 99 m-Tc perfusion scintigraphy and show reliable ability to judge perfusion intraoperatively.

Surgeons report ease of use with high contrast of green on gray background when using the Firefly setting on the DaVinci surgical robot [15]. The option of finger clutch allows for efficient inspection when judging perfusion, without potentially contaminating the operative field during the rest of the operation, a notable downside reported of using methylene blue. Another advantage reported was that, due to the high contrast, indocyanine green dye has an immediate effect and allows for perfusion to be judged rapidly [16]. A downside of the aforementioned was reported to be the requirement of quick judgment, as waiting too long possibly leads to ICG saturating the tissues resulting in a lack of contrast when judging perfusion, which is why timing is crucial. A drawback of using ICG is the need for the right dosing and concentration, as well as optimal settings on the DaVinci Firefly mode, in order to achieve the right level of fluorescence to judge perfusion. When this is not optimal it will lead to over or underestimation of effect.

A critical example of ease of use and reliability is the case where no extrahepatic perfusion was seen when tested with methylene blue dye, however when tested with ICG a small accessory artery to the common hepatic artery was seen and coagulated. Although this is a single observation, it shows the potential benefits of using ICG instead of methylene blue.

A limitation of our findings is the limited number of patients and all patients receiving both methylene blue and ICG, not allowing for proper comparison of outcome. In order to better compare outcomes, more research will have to be done.

In 2021, Spaggiari et al. published the first case report documenting the utilization of indocyanine green in a single patient undergoing HAIP placement [11]. This is the first multicenter case series showing reliability and feasibility of combining this procedure with the use of indocyanine green dye, confirming the findings by Spaggiari et al. In order to prove superiority a larger and prospective cohort is required and the use of ICG will have to be further optimized for perfusion testing.

In conclusion, ICG fluorescence poses a safe and reliable tool for intraoperative perfusion testing in placement of HAIP chemotherapy pumps. It is not possible, however, to conclude superiority for ICG based on the results of this study alone. Further research is required to definitively replace methylene blue.

## Supplementary Information

Below is the link to the electronic supplementary material.
